# Mesenteric lymph node cells from neonates present a prominent IL-12 response to CpG oligodeoxynucleotide via an IL-15 feedback loop of amplification

**DOI:** 10.1186/1297-9716-42-19

**Published:** 2011-02-02

**Authors:** Stéphanie Ferret-Bernard, Sonia Lacroix-Lamandé, Aude Remot, Coralie Metton, Nelly Bernardet, Bernard Charley, Françoise Drouet, Fabrice Laurent

**Affiliations:** 1 INRA, UR1282 Infectiologie Animale et Santé Publique, Equipe « Contrôle et Immunologie des Maladies Entériques du Nouveau-Né », Nouzilly, France; 2 INRA, UMR 1079 Systèmes d'Elevage, Nutrition Animale et Humaine, Equipe « Nutrition périnatale et adaptabilité intestinale », Saint Gilles, France; 3 INRA, UR0892 Unité Virologie et Immunologie Moléculaires, Jouy-en-Josas, France

## Abstract

At birth, the immune system is still in development making neonates more susceptible to infections. The recognition of microbial ligands is a key step in the initiation of immune responses. It can be mimicked to stimulate the immune system by the use of synthetic ligands recognising pattern recognition receptors. In human and mouse, it has been found that neonatal cytokine responses to toll-like receptor (TLR) ligands differ in many ways from those of adults but the relevant studies have been limited to cord blood and spleen cells. In this study, we compared the responses in neonate and adult sheep to CpG oligodeoxynucleotides (ODN), a TLR9 ligand, in both a mucosal and a systemic organ. We observed that in response to CpG-ODN more IL-12 was produced by neonatal than adult sheep cells from mesenteric lymph nodes (MLN) and spleen. This higher IL-12 response was limited to the first 20 days after birth for MLN cells but persisted for a longer period for spleen cells. The major IL-12-producing cells were identified as CD14^+^CD11b^+^. These cells were poor producers of IL-12 in response to direct stimulation with CpG-ODN and required the cooperation of other MLN cells. The difference in response to CpG-ODN between neonates and adults can be attributed to both a higher proportion of CD14^+^CD11b^+ ^cells in neonate lambs and their higher capacity to produce IL-15. The IL-15 increases IL-12 production by an amplifying feedback loop involving CD40.

## Introduction

Immune responses in neonates differ from those in adults due to differences in the relative proportions, phenotypes and functional properties of their immune cells [1-4]. In infant and neonate mouse a Th2 bias has been reported that leads to a reduced capacity to respond efficiently to vaccines that rely on a Th1 immune response for their efficacy. Immunoprophylactic strategies have therefore to be adapted for neonates and properly targeted. Pattern recognition receptors are expressed by cells of the innate immune system and identify microbial components or cellular stress. Toll-like receptors (TLR) belong to this family of receptors, and are attractive targets for immunostimulation strategies; consequently, many synthetic molecules that mimic bacterial or viral components have been generated. Synthetic CpG oligodeoxynucleotides (CpG-ODN) resembling bacterial DNA have been extensively used to promote Th1 immune responses [5] and to control both systemic and mucosal infections. We observed that a single administration of CpG-ODN to neonate mice can greatly reduce infection by *Cryptosporidium parvum *[6] by inducing the production IFNγ, a cytokine central to the control of this zoonotic parasite infecting intestinal epithelial cells [7,8]. CpG-ODN have also been shown to be safe to use in veterinary species, [9,10] and effective in ruminants for controlling bacterial [11,12], parasitic [13] and viral infections [14]. The potential of CpG-ODN for stimulating innate immune responses has been also demonstrated in neonate lambs in a study by Nichani et al. reporting that their administration can reduce viral shedding of bovine herpes virus-1 [15].

The specificities of the responses of human and mouse neonatal cells have been described. However, the relevant studies were limited to human cord blood cells and mouse spleen cells. Neonate small ruminants, being much bigger than rodent animal models, allow the recovery of large numbers of cells from various tissues facilitating investigations. In addition, data obtained in human or mouse cannot be directly extrapolated to veterinary species despite the conservation of TLR throughout evolution. This is because TLR responses to their agonists may differ between species due to differential expression among immune cell populations or differences in binding or signalling [16-18]. Exploiting the advantages of a large animal model, the goats, we previously investigated the cytokine response to various TLR ligands of cells isolated from neonatal and adult lymph nodes draining the intestine. The intestine is subjected to many changes after birth due to exposure to dietary antigens and colonization by the commensal flora. In response to TLR stimulation, neonate mesenteric lymph nodes (MLN) cells presented a stronger IFNγ and IL-12 response than their adult counterparts [19]. Although CD8^+ ^lymphocytes were identified as being responsible for the IFNγ production, the precise nature of the cells secreting IL-12 was not identified.

Using lambs as a model, we describe further investigations regarding the age-related differences of cytokine responses to TLR ligands. In particular, we aimed to determine until what age neonate MLN and spleen cells continued to produce more IL-12 than their adult counterparts in response to CpG-ODN stimulation and the reasons for the difference.

## Materials and methods

### Animals and cell isolation

The Préalpes adult sheep (aged 6 ± 1 year), neonates (aged 6 to 14 days) and lambs (aged 20 days) used were reared in conventional but protected sanitary facilities (PFIE, INRA, F-37380 Nouzilly, France). Newborn lambs were not separated from their mothers until one day after birth, to allow them to suckle colostrum. They were then fed *ad libitum *with reconstituted milk. Experimental protocols were designed in compliance with French law (Décret 2001-464 29/05/01) and EEC regulations (86/609/CEE) concerning the care and use of laboratory animals. Euthanasia was performed after electric stunning according to AMVA guidelines (2007) on euthanasia. Cells from freshly removed MLN or spleen were isolated as previously described [19].

### Reagents

CpG-2006 5'TCGTCGTTTTGTCGTTTTGTCGTT3' and control-CpG-2006 (Ctl-ODN) 5'TGCTGCTTTTGTGCTTTTGTGCTT3' have a phosphorothioate backbone and were purchased from Sigma-Aldrich (Lyon, France). Recombinant human TGFβ1 and recombinant type I interferons (IFN) (IFNα hybrid, constructed with human IFN αA and αD is active in all mammalian species) were obtained from AbD Serotec (Oxford, UK). Recombinant human IL-15 was from Immunotools (Friesoythe, Germany). Recombinant ovine IL-12 and IL-10 were kindly provided by S. Wattegedera (Moredun Research Institute, Edinburgh, UK). Anti-CD40 mAb supernatant clone ILA156 originally produced by J. Naessens (ILRAD, Nairobi, Kenya) was provided by I. Schwartz-Cornil.

### CpG-ODN stimulation and cytokine quantification by ELISA and by bioassay

Mesenteric lymph node (MLN) or spleen cells were stimulated for 48 h, at a density of 1.5 × 10^6^/mL in complete RPMI 1640 medium (Gibco-Invitrogen, Cergy-Pontoise, France) supplemented with 10% FCS, 100 IU/mL penicillin, 100 μg/mL streptomycin sulphate and 50 μM β-mercaptoethanol (Merck Chemicals Ltd., Nottingham, UK) with Ctl-ODN (1 μM) or CpG-2006 (1 μM). In the IL-12 neutralisation assay, mouse anti-bovine IL-12 (clone CC301, Serotec) or its isotype control (mouse IgG2a, Caltag-Invitrogen) was added, at a concentration of 10 μg/mL, at the same time as the TLR agonist.

In some experiments, rhTGFβ1, rovIL-10, rh type I IFN or rhIL-15 was added to MLN cells at the same time as Ctl-ODN or CpG-2006. Culture supernatants were harvested and stored at -20°C until assayed for the detection of cytokines by ELISA. Intracellular staining for IL-12 (clone CC301, AbD Serotec) was performed on MLN cells cultured in complete RPMI medium with Ctl-ODN or CpG-2006 for 25 h. Brefeldin A (Sigma) was added to the cells at a concentration of 5 μg/mL, for the last 5 h of culture.

Pairs of antibodies against bovine cytokines were obtained from AbD Serotec; these antibodies have been shown to recognise ovine IL-12 [20,21], IFNγ [21] and IL-10 [20,22]. ELISA was carried out as previously described [19]. Incubations were performed at 37°C for IL-10 ELISA and at room temperature for the other ELISA. To assess biologically active TGFβ1 secretion by ovine cells, we used the TGFβ1 Emax^® ^ImmunoAssay System (Promega, Charbonnières-les-Bains, France) according to the manufacturer's instructions.

Type I IFN in cell supernatants were quantified using a cytopathic effect reduction assay with Madin-Darby bovine kidney cells challenged with vesicular stomatitis virus. An internal recombinant IFNα reference was included as described elsewhere [23]. Each supernatant was tested over eight serial dilutions. Results are expressed as type I IFN units per mL.

### Colistin treatment and Gram negative bacterial enumeration

Control and colistin-treated animals were separated from their mothers directly after birth and bottle-fed every 12 h for the first day with preheated ewe colostrum containing or not containing 50 000 IU/kg body weight of colistin (Virbac, Carros, France). Thereafter, they were fed daily *ad libitum *with reconstituted milk containing or not containing 100 000 IU/kg body weight of colistin (Virbac) until the age of 20 days. Mean body weights were used to prepare the milk containing colistin: 3 kg for 2- to 8-day-old, 4 kg for 9- to 14-day-old and 7 kg for 15- to 20-day-old lambs. Lambs were slaughtered at age 20 days and both MLN (for in vitro cell stimulation as previously described) and distal ileum contents were collected. To determine the total number of Gram -ve bacteria, serial dilutions of ileal content homogenised in PBS were plated onto Drigalski lactose agar (selective medium for the isolation of Gram -ve bacteria). The plates were incubated overnight at 37°C and colonies counted.

### Cell sorting and flow cytometry

A high-speed MoFlo cell sorter (Dako, Trappes, France) was used to sort freshly isolated total MLN cells. In these experiments, cells were labelled with a mouse antibody specific for ovine CD14 (clone CAM36A, VMRD, Pullman, USA) and stained with a fluorochrome-conjugated goat anti-mouse immunoglobulin antibody (Caltag-Invitrogen). Lymph granulocytes in sheep display intermediate levels of CD14 expression [24]. CD14^+ ^cell sorting was therefore always performed after gating on non-granulocytic MLN cells according to SSC/FSC analysis, although most granulocytes were removed on the Histopaque gradient. Both CD14^+ ^and CD14^- ^cell fractions were cultured in vitro, with Ctl-ODN or CpG-2006, or were directly analysed by qRT-PCR.

For intracellular staining of IL-12, stimulated cells were cultured in the presence of Brefeldin A for the last 5 h. First, the following purified monoclonal antibodies directed against ovine markers were used for surface staining: CD11b (clone MM12A, VMRD), CD14 (clone CAM36A, VMRD), MHC class II (clone 28.1, AbD Serotec) and CD205 (clone CC98, AbD Serotec). Fixed and permeabilised cells were then incubated with the mouse anti-bovine/ovine IL-12 antibody (clone CC301, AbD Serotec).

### Electron microscopy

Sorted CD14^+ ^cells were fixed, post-fixed, dehydrated in a graded series of ethanol solutions and embedded in Epon resin (Sigma). Ultrathin sections were cut, stained and examined under a Jeol 1010 transmission electron microscope (Jeol, Croissy-sur-Seine, France).

### RNA isolation and real-time RT-PCR

RNA was extracted with the NucleoSpin RNA II kit (Macherey-Nagel, Hoerdt, France), according to the manufacturer's instructions and quantified using Nanodrop (Thermo Fisher Scientific, Courtaboeuf, France). Purified RNA was reverse-transcribed using oligo(dT) primers and M-MLV reverse transcriptase (Promega). Primer pairs were designed using Primer 3 software (Additional file 1, Table S1). Each primer was designed on different exons to span the intervening intron and thus avoid amplification from contaminating genomic DNA. For each primer pair, all qPCR displayed an efficiency of between 90% and 110%. Diluted cDNA was combined with primers and IQ SYBRGreen Supermix (Bio-Rad, Hercules, USA) according to the manufacturer's recommendations and real-time assays were run on a Bio-Rad Chromo 4 (Bio-Rad). The specificity of the qPCR reactions was assessed by analysing the melting curves of the products and size verification of the amplicons. To minimise sample variations, we used identical numbers of cells and high quality RNA. Hypoxanthine phosphoribosyltransferase (HPRT) mRNA levels were used to normalise RNA quantification.

### Statistical analyses

Non-parametric Mann-Whitney (two groups) and Kruskal-Wallis (three or more groups) statistical tests were used to compare unpaired values. In case of paired values, paired *t*-tests were performed.

## Results

### IL-12 and IFNγ responses of neonatal and adult MLN cells to CpG-ODN stimulation

**Figure 1:**
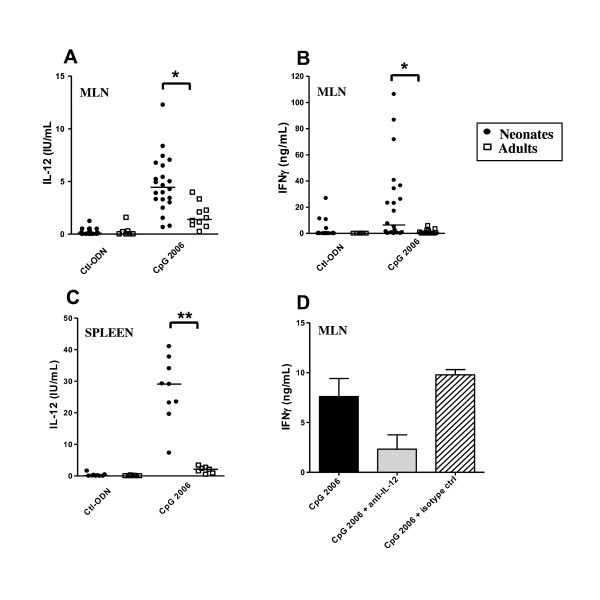
**Comparative IL-12 and IFNγ responses of neonatal and adult cells following TLR agonist stimulation**. Neonatal and adult MLN (A, B) and spleen cells (C) were cultured in vitro with 1 μM Ctl-ODN or 1 μM CpG-2006. Supernatants were harvested after 48 h of culture and ELISA was carried out for IL-12 (A, C) and IFNγ (B) secretions. (D) Mouse anti-bovine IL-12 (grey bar) or its isotype control (hatched bar) was added to neonatal MLN cells, at the same time as CpG-2006 or Ctl-ODN. IFNγ secreted into the culture supernatants was determined after 48 h. The mean ± SEM concentration of IFNγ secretion is shown (CpG-2006 - Ctl-ODN). * p ≤ 0.005; ** p ≤ 0.001.

Neonatal MLN cells stimulated in vitro with CpG-2006 produced variable but significantly larger amounts of IL-12 (Figure 1A) and IFNγ (Figure 1B) than adult MLN cells. This higher IL-12 response was also observed for neonatal spleen cells (Figure 1C). Adult MLN and spleen effector cells (T cells and NK cells) were not intrinsically impaired for IFNγ production compared to neonatal cells, as they produced significantly larger amounts of this cytokine in response to non-specific stimulation in vitro with phorbol myristate acetate associated with ionomycin (data not shown). Neutralisation of IL-12 in vitro indicated that the higher production of IFNγ by neonatal MLN cells was due in large part to the higher level of IL-12 released upon CpG-2006 stimulation (Figure 1D, 78 ± 12% inhibition, p < 0.005). We therefore investigated the particular features of the neonatal cells producing IL-12 in response to CpG-2006.

### Age-dependent IL-12 responses of MLN and spleen cells to CpG-ODN stimulation

**Figure 2:**
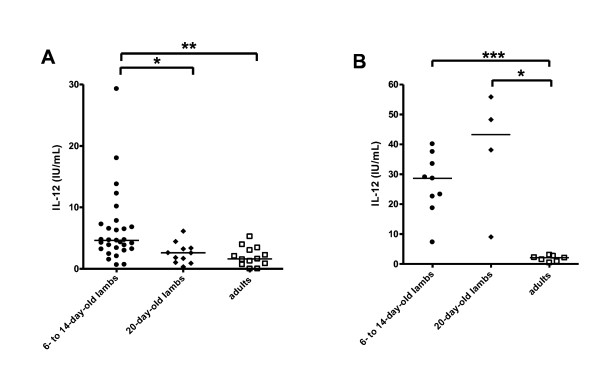
**Age-dependent IL-12 responses**. MLN and spleen cells from 6- to 14-day-old neonates (closed circles), 20-day-old lambs (closed diamonds) and adults (open squares) were cultured in vitro for 48 h, with 1 μM CpG-2006 or 1 μM Ctl-ODN. At the end of the culture period, supernatants were harvested and ELISA carried out to assay IL-12 secreted by MLN cells (A) or spleen cells (B). Medians are shown for each stimulus. * p ≤ 0.01; ** p ≤ 0.001; *** p ≤ 0.0005.

We first studied the evolution with age of the stronger response in vitro of neonatal cells. We measured IL-12 secretion by MLN and spleen cells from 20-day-old lambs, neonates and adults. IL-12 secretion by MLN cells in response to in vitro CpG-2006 stimulation rapidly decreased with age: cells from 20-day-old lambs producing as little IL-12 as adult cells (Figure 2A). By contrast, splenocytes from 20-day-old animals, like neonatal cells, secreted significantly larger, albeit variable, amounts of IL-12 than adult cells (Figure 2B). Thus, the higher IL-12 response began to decline earlier in MLN cells than in spleen cells, suggesting major changes in TLR responsiveness in the lymph nodes within the first few days of life.

### Effect of Gram negative intestinal flora on the response of neonatal MLN cells to CpG-ODN

**Figure 3:**
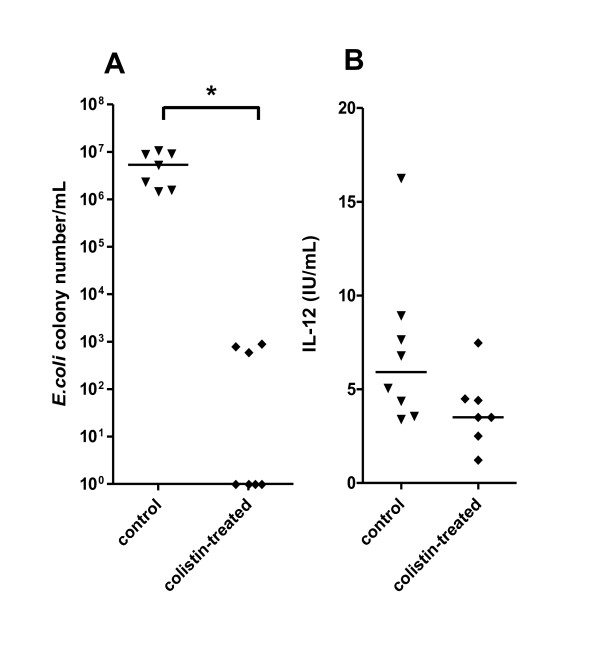
**Effect of antibiotic treatment of lambs on the IL-12 response of MLN cells to CpG-2006**. Lambs were treated daily for the first 20 days of life with colistin (closed diamonds) or not treated (closed triangles). Gram -ve colony counts per mL of distal ileum content from the two groups were calculated as described in the methods (A) and IL-12 secretion of MLN cells in response to CpG-2006 (CpG-2006 - Ctl-ODN) was determined after 48 h of culture by ELISA (B). * p ≤ 0.001.

The decrease of IL-12 secretion between 14 and 20 days after birth could be related to progressive installation of the intestinal microflora. Gram -ve commensal bacteria have a large impact on the formation of intestinal lymphoid tissues and the establishment of gut homeostasis in mice [25]. To assess the possible role of Gram -ve bacteria colonisation in our model, lambs were fed with milk supplemented with the antibiotic colistin from birth to 20 days of age. The Gram -ve bacterial count in the distal ileal content of these animals was at least 4 log lower than in controls (Figure 3A) but IL-12 response to CpG-2006 was not significantly different (Figure 3B). This suggests that another mechanism was responsible for the higher IL-12 secretion by neonatal MLN cells upon TLR ligation.

### IL-12-producing cells among lamb bulk MLN cells

**Figure 4:**
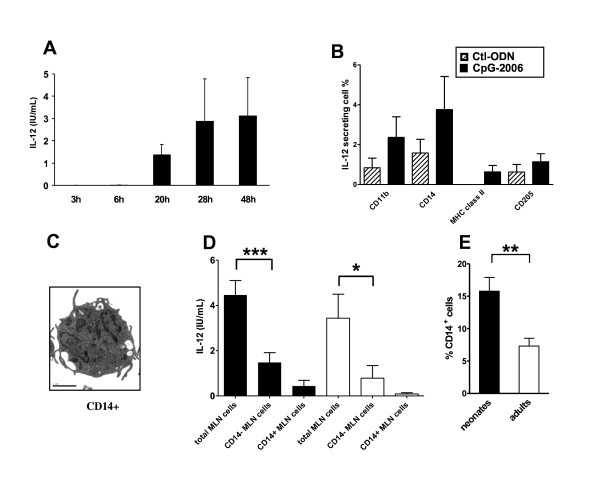
**Characterisation of IL-12-producing cells**. Kinetics of the IL-12 response to CpG-2006 during the first 48 h was followed by ELISA. Mean ± SEM IL-12 secretion (CpG-2006 - Ctl-ODN) is shown for each time point. Means ± SEM were calculated from three independent experiments (three different neonates) giving similar results (A). Neonatal MLN cells were cultured in vitro with 1 μM Ctl-ODN (hatched bars) or 1 μM CpG-2006 (black bars) for 25 h, with brefeldin A added for the last 5 h. IL-12 intracellular staining was combined with labelling of CD11b, CD14, MHC class II and CD205. Values reported are means ± SEM from three independent experiments (three different neonates) giving similar results (B). Neonatal CD14^+ ^MLN cells were observed by transmission electron microscopy. The scale bar on the representative picture indicates 2 μm (C). Neonatal (n = 11, closed bars) and adult (n = 6, open bars) total MLN cells and sorted CD14^- ^cells and CD14^+ ^cells (n = 6 for neonates and n = 5 for adults) were stimulated for 48 h with 1 μM CpG-2006 or 1 μM Ctl-ODN and ELISA was used to assay secreted IL-12 (CpG-2006 - Ctl-ODN) (D). Mean ± SEM of CD14^+ ^cell proportions in 6- to 14-day-old neonatal (n = 27, closed bar) and adult (n = 13, open bar) total MLN cells were assessed by single staining and flow cytometry (E).* p ≤ 0.05; ** p ≤ 0.01; *** p ≤ 0.001.

We aimed to identify the major IL-12 secreting cell population to characterise more precisely the mechanisms governing the differences between neonates and adults. ELISA kinetic assays showed that IL-12 secretion started between 6 and 20 h following CpG stimulation and that the secretion doubled between 20 h and 28 h post-stimulation (Figure 4A). We stained neonatal MLN cells for intracellular IL-12 at 20 h post-stimulation. The bar graph (Figure 4B) displays the percentage of IL-12 secreting cells among CD11b, CD14, MHC class II and CD205 positive cells. CD14^+ ^cells but also CD11b^+ ^cells were major contributors to IL-12 secretion in response to CpG. Flow cytometry analysis showed that most of the CD14^+ ^cells co-expressed CD11b. Moreover, all CD11b^+ ^cells co-expressed CD14 (data not shown). We therefore decided to focus our study on CD14^+ ^cells. Transmission electron microscopy revealed that neonatal CD14^+ ^MLN cells had numerous processes and horseshoe-shaped nuclei, as expected for cells of the myeloid lineage (Figure 4C). We also observed several dark electron-dense granules and a well developed endosomal compartment resembling that of ovine monocyte-derived dendritic cells (DC) [26] and a CD11b^int/high ^subpopulation of bone marrow-derived DC [27].

CD14^**+ **^and CD14^- ^cells purified by flow cell sorting from neonate and adult MLN were stimulated with CpG-2006. The absence of CD14^+ ^cells inhibited greatly IL-12 secretion not only in neonate (67 ± 7%) but also in adult (85 ± 8%) samples, indicating the essential role of these cells in both groups (Figure 4D). Some CD14^- ^cells participated in the residual IL-12 secretory activity (33 ± 7%) in neonates and (15 ± 8%) in adults. We cultured the same numbers of neonate or adult sorted CD14^+ ^cells in the presence of CpG for 48 h but very little or no IL-12 was detected. Therefore, CD14^+ ^are poor IL-12-producing cells in the absence of CD14^- ^cells, suggesting an indirect activation mechanism (Figure 4D). Flow cytometry analyses also revealed that the proportion of CD14^+ ^cells among bulk MLN cells was twice as high in neonates as in adults (Figure 4E). Although we did not observe a direct correlation between the proportion of CD14^+ ^cells in the MLN and the IL-12 response to CpG-2006 (data not shown), this quantitative difference may nevertheless contribute to the higher responsiveness of neonatal MLN cells to CpG-ODN.

### Effects of TGFβ1, IL-10 and type I IFN on the cytokine response to CpG-ODN

**Figure 5:**
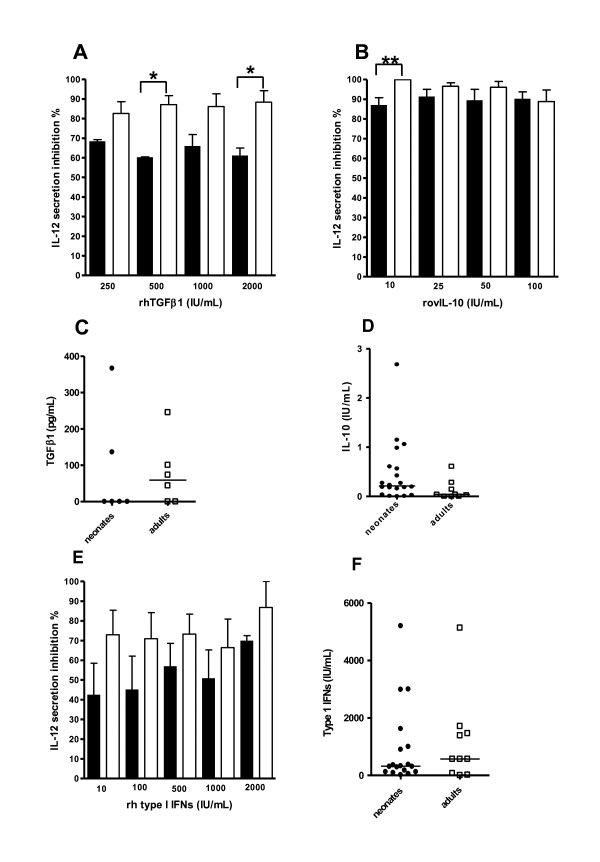
**Effect of regulatory cytokines on the IL-12 response to CpG-2006**. MLN cells from neonates (n = 3-5, closed bars) and adults (n = 5-7, open bars) were stimulated in vitro for 48 h with 1 μM of CpG-2006 or 1 μM of Ctl-ODN in the presence of each of various amounts of rhTGFβ1 (A), rovIL-10 (B) and rh type I IFN (E) and IL-12 secreted by MLN was assayed by ELISA (CpG-2006 - Ctl-ODN). The percentage of IL-12 secretion inhibition is shown for each dose of recombinant cytokine. MLN cells from neonates (closed circles) and adults (open squares) were cultured in vitro for 48 h, with 1 μM CpG-2006 or 1 μM of Ctl-ODN. TGFβ1 (C) and IL-10 (D) production was measured by ELISA and type I IFN activity by bioassay (F). Plot values were calculated by subtracting the values for Ctl-ODN stimulated cells from those for CpG-2006 stimulated cells. Medians are indicated for each group. * p ≤ 0.05; ** p ≤ 0.005.

Immune cells in the intestinal mucosa are conditioned by many regulatory cytokines, including TGFβ1 and IL-10, helping to avoid responses against commensal flora and dietary antigens. Booth et al. observed the presence of regulatory B cells producing large amounts of IL-10 in sheep Peyer's patches and downregulating TLR9-induced cytokine responses [28]. Therefore, we tested the effect of rhTGFβ1 and rovIL-10, during CpG-ODN stimulation, on the IL-12 response of adult and neonate MLN cells. TGFβ1 and IL-10 each strongly inhibited IL-12 release in both neonates and adults (Figures 5A, B). At some concentrations, IL-12 secretions in response to CpG from adult MLN cells were significantly more sensitive than neonate MLN cells to these regulatory cytokines. This may result from a higher expression of the TGFβ1 and IL-10 receptors on adult MLN cells compared to those of neonates and/or from intracellular signalling differences. Possibly, regulatory T cells, producing TGFβ1 and IL-10, are more numerous among adult MLN cells, explaining the lower overall IL-12 secretion by total MLN cells. However, analysis of FoxP3 mRNA expression by MLN cells was not consistent with this possibility, as the expression level was similar in neonatal and adult samples (data not shown). Besides, no significant differences related to the secretion of these two regulatory cytokines following CpG stimulation were observed between neonates and adults (Figures 5C, D). However, when TGFβ1 secretion was detected in response to CpG-ODN, it may have affected preferentially the IL-12 response of adult animals.

We next searched for differences that may affect the signal provided by CD14^- ^cells to CD14^+ ^cells for IL-12 production. It has been reported that type I IFN either stimulate or inhibit IL-12 production by myeloid cells [29,30]. TLR9-triggering of plasmacytoid DC (pDC) induces the secretion of large amounts of type I IFN [31]. We investigated exactly how type I IFN influence IL-12 secretion by CD14^+ ^MLN from neonates and adults stimulated by CpG-2006. Interestingly, rhIFN (IFNα hybrid) displayed suppressive properties on both neonate and adult MLN cells (Figure 5E). We performed a type I IFN bioassay on supernatants from neonate and adult MLN cells after CpG-2006 stimulation to test whether the lower IL-12 response in adults was associated with higher levels of type I IFN, but no difference in type I IFN concentrations were observed (Figure 5F).

### Role of IL-15 in the IL-12 response of MLN cells to CpG-ODN

**Figure 6:**
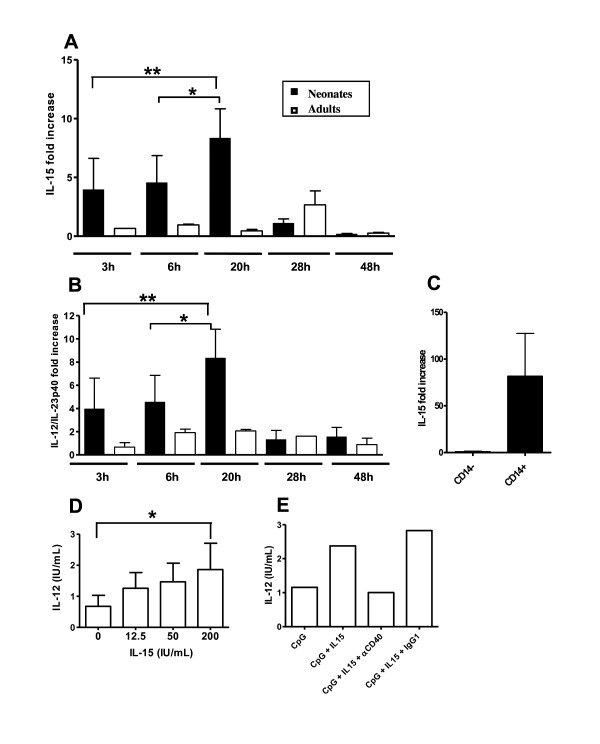
**Role of IL-15 in the IL-12 response of MLN cells to CpG-2006**. The kinetics of IL-15 (A) and IL-12/IL-23p40 (B) mRNA production in response to CpG-2006 was determined by quantitative RT-PCR. Experiments were performed with MLN cells isolated from three neonates and three adults. 20 h post-stimulation, MLN cells isolated from three different neonates were recovered and sorted into CD14^- ^and CD14^+ ^cell subsets and IL-15 mRNA was assayed by quantitative RT-PCR in each subset (C). After normalisation with the reference gene (HPRT), the RT-PCR data are reported as means ± SEM values of the *ratio *of CpG-2006 stimulated to Ctl-ODN stimulated cells (A, B, C). MLN cells, isolated from eight adults, were stimulated with CpG-2006 for 48 h, with or without various amounts of rhIL-15; secreted IL-12 was measured by ELISA (D). The mean ± SEM level of IL-12 secretion is shown for each dose of recombinant cytokine (CpG-2006 - Ctl-ODN). Adult MLN cells were stimulated with CpG-2006 (or Ctl-ODN) and 200 IU/mL of IL-15 in the presence of anti-CD40 mAb or its isotype control and IL-12 was quantified by ELISA 48 h later (CpG-2006 - Ctl-ODN). Data are from one representative animal of five, all giving similar results (E). * p < 0.05; ** p < 0.005.

A feedback loop of amplification has been described for IL-15 in the IL-12 response to CpG-ODN involving CD40/CD40L interaction [32]. As no ELISA is yet available for sheep IL-15, we studied its mRNA by quantitative RT-PCR. qRT-PCR with MLN cells stimulated with CpG showed that IL-15 mRNA was much more abundant in neonatal cells than in their adult counterparts, from 3 h to 20 h after stimulation with a kinetic of expression similar to that of the IL-12/23p40 chain (Figures 6A, 6B). To identify the main source of IL-15-producing cells we analysed IL-15 mRNA in CD14^+ ^and CD14^- ^cells from neonates activated with CpG-2006: IL-15 mRNA was more abundant in CD14^+ ^cells (Figure 6C). To test that IL-15 can potentiate IL-12 secretion in adult MLN cells, we added rhIL-15 at the same time as CpG and the result was an IL-12 secretion that was about three-fold higher than in the controls (Figure 6D). We next analyzed the involvement of CD40 in the IL-12 production by adding an anti-CD40 mAb concomitantly with IL-15 and CpG-2006 to adult MLN cell culture. CD40 binding by the antibody reduced by 51 ± 19% the IL-12 response relative to the isotype control mAb (Figure 6E). This suggests that CD40 binding by the mAb limits its interaction with its ligand.

## Discussion

Neonates are more sensitive to infections in particular when exposed to poor sanitary conditions before their immune system is fully developed. It is therefore important to develop immunoprophylactic strategies dedicated to neonates. With the discovery of TLRs and their importance in the initiation of immune responses to invading pathogens, a large set of new synthetic adjuvants has been developed including CpG-ODN for use against infectious disease and cancer [33-35]. In human and mouse, the age-dependent response to TLR has only been documented in cord blood cells and spleen cells, respectively [36-38]. The size of small ruminants facilitates investigation of these responses in different organs including mucosal tissues and their draining lymph nodes [19,28].

Here, we report that cells isolated from the MLN and spleen in neonate lambs produced more IL-12 than their equivalents in adults in response to CpG-2006. This higher IL-12 production was responsible in large part for the higher IFNγ response in neonatal stimulated MLN cells as revealed by in vitro IL-12 neutralisation assay. We next examined the age until which neonate cells produced more IL-12 than adults and found that this stronger IL-12 response was restricted to about the first two weeks of life: from age 20 days MLN from lambs produced similar amounts of IL-12 as those from adults. Interestingly, spleen cells from 20-day-old lambs continued to produce more IL-12 in response to CpG-ODN suggesting that a rapid and specific change in TLR agonist responsiveness occurs in MLN. During this period, the dietary regimen of lambs was similar with reconstituted milk provided *ad libitum*. As MLN drain the intestinal tissue, we thought that the progressive installation of the commensal flora might have influenced TLR9 responsiveness. Indeed, Gram -ve bacteria are important for lymphoid tissue development [25]. We set up a protocol to control Gram -ve bacteria by feeding lambs with reconstituted milk containing the antibiotic colistin. Although dramatically reducing Gram -ve bacterial counts in the intestine of 20-day-old lambs, this treatment did not significantly affect the capacity of MLN cells to produce IL-12. We next investigated the role of regulatory cytokines, including IL-10 and TGFβ1 produced in large amounts in intestinal tissue. These cytokines effectively suppressed IL-12 production by MLN cells stimulated with CpG-2006. Although, in response to CpG-2006 stimulation in vitro, they were produced in similar amounts by MLN cells whatever the age of the animal and only detected for some animals. Adult MLN cells activated with CpG-ODN presented a slightly higher sensitivity to these cytokines at certain concentrations. Therefore we cannot exclude that for some animals it may participate to the lower IL-12 response of adults. Booth et al. [28] identified a CD5^-^CD21^**+ **^B cell population in the ileal Peyer's patches (iPP) in adult sheep. These cells reduce the expression of IFNγ and IL-12 by iPP cells stimulated with CpG-ODN by producing large amount of IL-10. However, in agreement with our results the authors did not observe any similar regulatory mechanism in the MLN [28]. To continue, we needed to identify the cell population responsible for IL-12 production. Intracellular IL-12 staining revealed that cells expressing CD14 and CD11b were the main producers. These cells have a dendritic morphology and resemble sheep bone marrow-derived DC [26,27]. Possibly, these myeloid cells originate from blood monocytes and differentiate into inflammatory DC. These DC are known to produce abundant IL-12 and potently stimulate Th1 responses [39]. We observed that the percentage of CD14^+ ^cells was higher in MLN of neonates than of adults. This might contribute to the higher IL-12 response of neonate MLN but we did not observe a direct correlation between the percentage of CD14^+ ^cells in MLN and the level of IL-12 produced after CpG stimulation; consequently, we do not think that this is the complete explanation. CD14^+ ^cells respond poorly to direct CpG-2006 stimulation but require the cooperation of other cells to release IL-12. It has been reported that *Mycobacterium bovis*-induced IL-12 secretion by bovine DC is enhanced by WC1^+ ^γδ T cells [40]. Moreover, Hedges et al. observed that bovine γδ T cells express different TLR including TLR9 and respond directly to some pathogen-associated molecular patterns like LPS and peptidoglycan, however in this study the response of γδ T cells to CpG-ODN was not investigated [41]. Therefore in our experimental model γδ T cells may cooperate with CD14^+ ^cells for the IL-12 response to CpG-ODN. Another population, pDC have been shown to cooperate with the IL-12 response of conventional DC (cDC) to CpG-ODN [42]. Activated pDC produce alpha IFN, cytokines that in our experimental model inhibited the IL-12 response of MLN cells stimulated with CpG-2006. After stimulation, we found only a low level of type I IFN activity. This is probably because CpG-2006 is a class B-ODN and this class induces type I IFN much more weakly than do class A- and class C-ODN. However, the level of type I IFN activity released by neonates and adults being equivalent, this cannot explain the difference in IL-12 production observed. Kuwajima et al. showed that CpG-treated IL-15-deficient mice produced little IL-12 [32]. They observed that CpG-stimulated cDC were the main producers of both IL-15 and IL-12, but cDC did not produce IL-12 in the absence of pDC. After CpG-2006 stimulation, we observed a much stronger concurrent upregulation of IL-12p40 and IL-15 mRNA in MLN cells from neonate than adult sheep suggesting that in adults IL-15 availability may be insufficient for the full activation. We therefore added exogenous IL-15 to CpG-stimulated adult MLN cell and observed an increase in IL-12 production of up to a three-fold. When a CD40 mAb was added to the culture medium, it reduced IL-12 production by half. This is consistent with the findings of Kuwajima et al. [32] who observed that IL-15-induced CD40 expression by cDC and interaction between CD40 on cDC and CD40 ligand on pDC led to IL-12 production by cDC. IL-15 therefore plays a key role in the innate response to CpG and seems to act via a feedback amplification loop. We recently demonstrated that IL-15 is also an important molecule for NK cells in neonates, another innate immune cell population [3]. Indeed, NK cells from one week-old neonate calves expanded in presence of IL-15, but not IL-2, presented both a higher cytotoxicity than their equivalents from older animals in direct lysis assay and a higher IFNγ response to IL-12 when associated with NKp46 receptor stimulation.

Here, we demonstrate the potential of CpG-ODN to induce a preferential Th1-type cytokine and IL-15 response in neonate lambs. CpG oligonucleotides are therefore potentially useful molecules for enhancing the efficacy of vaccines against intracellular pathogens affecting these animals.

## Competing interests

The authors declare that they have no competing interests.

## Authors' contributions

SFB conceived and designed the experiments, performed the experiments and the statistical analysis and wrote the manuscript. SLL performed experiments and the statistical analysis and corrected the manuscript. AR performed experiments. CM and NB performed the RNA isolation and real time RT-PCR experiments. BC carried out the type I IFN bioassays. FD performed the experiments. FL conceived, designed, performed and coordinated the experiments and wrote the manuscript. All authors read and approved the final manuscript.

## Supplementary Material

Additional file 1 **List of primers used for qRT-PCR analysis**. Table showing complete list of primer pairs used for qRT-PCR analysis. Click here for file
