# Supplementary material for: Mesenteric lymph node cells from neonates present a prominent IL-12 response to CpG oligodeoxynucleotide via an IL-15 feedback loop of amplification
Source: Vet Res. 2011 Feb 2;42(1):19. doi: 10.1186/1297-9716-42-19 (PMC3039596; doi:10.1186/1297-9716-42-19)
Supplement: Additional file 1 — List of primers used for qRT-PCR analysis. Table showing complete list of primer pairs used for qRT-PCR analysis. [file 1297-9716-42-19-S1.DOC]

**Table S1: Primers used for qRT-PCR analysis**
